# Temporary Flow Diversion in Oncological Embolization Procedures Using Degradable Starch Microspheres

**DOI:** 10.3390/diagnostics14242844

**Published:** 2024-12-17

**Authors:** Francesco Giurazza, Pierleone Lucatelli, Fabio Corvino, Renato Argirò, Pietro Roccatagliata, Anna Maria Ierardi, Raffaella Niola

**Affiliations:** 1 Vascular and Interventional Radiology Department, Cardarelli Hospital, Via A. Cardarelli 9, 80131 Naples, Italy; fabio.corvino@aocardarelli.it (F.C.); raffaella.niola@aocardarelli.it (R.N.); 2 Vascular and Interventional Radiology Unit, Department of Radiological, Oncological and Anatomo-Pathological Sciences, Sapienza University of Rome, 00185 Rome, Italy; pierleone.lucatelli@gmail.com; 3 Interventional Radiology Unit, Department of Biomedicine and Prevention, Azienda Ospedaliera Universitaria Tor Vergata, Viale Oxford 81, 00133 Rome, Italy; renato.argiro@gmail.com; 4 Department of Precision Medicine, University of Campania “L. Vanvitelli”, 80138 Naples, Italy; peterocca94@hotmail.it; 5 Radiology Department, Fondazione IRCCS Cà Granda, Ospedale Maggiore Policlinico, 20122 Milan, Italy; amierardi@yahoo.it

**Keywords:** flow diversion, embolization, temporary, oncological, degradable starch microspheres

## Abstract

Objectives: This study aims to report on the application of degradable starch microspheres to provide flow diversion by means of temporary embolization of healthy tissues in oncological endovascular procedures when tumor feeding vessels are not selectively accessible. Methods: This is a multicenter retrospective analysis of patients undergoing visceral embolization procedures of malignancies. The inclusion criteria were as follows: flow diversion performed by injection of degradable starch microspheres, visceral embolization procedures with unfeasible superselective catheterism of the target, and a malignant pathology. Technical success was defined as complete flow diversion with temporary exclusion of the non-target district from arterial flow, associated with successful embolization of the target. Clinical success was intended as procedural achievement with patient clinical improvement. Results: Sixteen patients were included in this analysis. Peripheral embolization procedures were performed in the coeliac visceral district all in oncologic patients, including 4 transarterial radioembolization work-up procedures in patients with hepatocarcinoma, 10 chemioembolization procedures in patients with hepatocarcinoma (8) or cholangiocarcinoma (2), and 2 palliative transarterial embolizations in patients with gastric cancer. Technical success was obtained in 100% of the cases, while clinical success was reached in 87.5%: in two chemioembolization procedures, despite technical success, the procedural clinical benefits were partial, with an incomplete target lesion response. Minor complications occurred in five patients (31.2%). Conclusions: In this study, temporary flow diversion with degradable starch microspheres during oncological embolization procedures was safe and effective; this approach is suitable to protect healthy surrounding tissues when vessels feeding the target cannot be selected with the microcatheter.

## 1. Introduction

Flow diversion in interventional radiology represents a technique to divert arterial flow from a vessel or a lesion. It has been a concept widely applied in the treatment of intracranial aneurysms for more than a decade [1], thanks to the development of specific metallic stents designed with flow diverter mash technology [2]. In peripheral extracranial interventions, flow diverter stents are similarly applied to repair visceral and renal aneurysms [3], though at a lower rate compared to the neurovascular environment because more alternative strategies are available.

Apart from flow diverter stents in visceral aneurysms, very few data are available about the possibility to divert arterial flow, especially when it comes to a temporary flow diversion during cancer embolization. It is not uncommon that during embolization procedures, non-target healthy tissues are sacrificed because the target is not selectively accessible, as for instance in complex liver cancer hypervascular arterial networks [4]. In this scenario, a solution could be to protect healthy tissues by means of temporary embolization in order to divert arterial flow to the target.

In light of this approach, Embocept-S^®^ (Pharmacept, Berlin, Germany) degradable starch microspheres (DSMs) can represent a useful tool: these are calibrated 50 μm (75%: 20–200 μm) microparticles with a half-life of 35 min and complete reabsorption in 90 min, thanks to their hydrolyzed potato starch polymer structure degraded by α-amilase. Currently, Embocept-S^®^ DSMs are approved for transarterial chemioembolization (TACE) of multiple liver cancers, acting as carriers for different chemiotherapic agents [5].

This study aims to report on the application of Embocept-S^®^ DSMs to provide flow diversion by means of temporary embolization of healthy tissues in oncological endovascular procedures when the tumor feeding vessels are not selectively accessible.

## 2. Materials and Methods

This is a multicenter retrospective analysis of patients undergoing visceral embolization procedures of malignancies by applying flow diversion to avoid non-target districts.

Electronic medical records and picture archive systems were reviewed from January to October 2024.

All endovascular treatments were indicated after local oncological multidisciplinary team meetings.

The inclusion criteria were as follows: flow diversion performed by injection of degradable starch microspheres, visceral embolization procedures with unfeasible superselective catheterism of the target, and malignant disease.

The exclusion criteria were as follows: previous surgery in the treated district and stenting of the target district.

The following parameters were investigated: sex, age, coagulation status (INR and platelets; antiplatelets/anticoagulants at the time of the procedure), vascular district, embolics delivered to the target, pre- and post-procedural laboratory values (liver function, blood count), and a 30-day CT follow-up.

Technical success was defined as complete flow diversion with temporary exclusion of the non-target district from arterial flow by means of DSM embolization, associated with successful embolization of the target, detected using digital subtracted arteriography (DSA) or ConeBeamCT scan acquisition.

Clinical success was defined as procedural achievement with patient clinical improvement.

Complications were recorded according to the CIRSE standard classification for complications [6].

### 2.1. Intervention

Patients signed a written informed consent before the procedure.

All interventions were planned on the basis of a preprocedural contrast-enhanced CT acquired within 30 days; patients had normal coagulation function (INR < 1.5; platelets > 50,000/μL) but 6 took up antiplatelet therapy at the time of the procedure.

Blood count and liver function were assessed before the procedure; in case of total bilirubin > 2 mg/dL, interventions were not performed.

**Figure 1:**
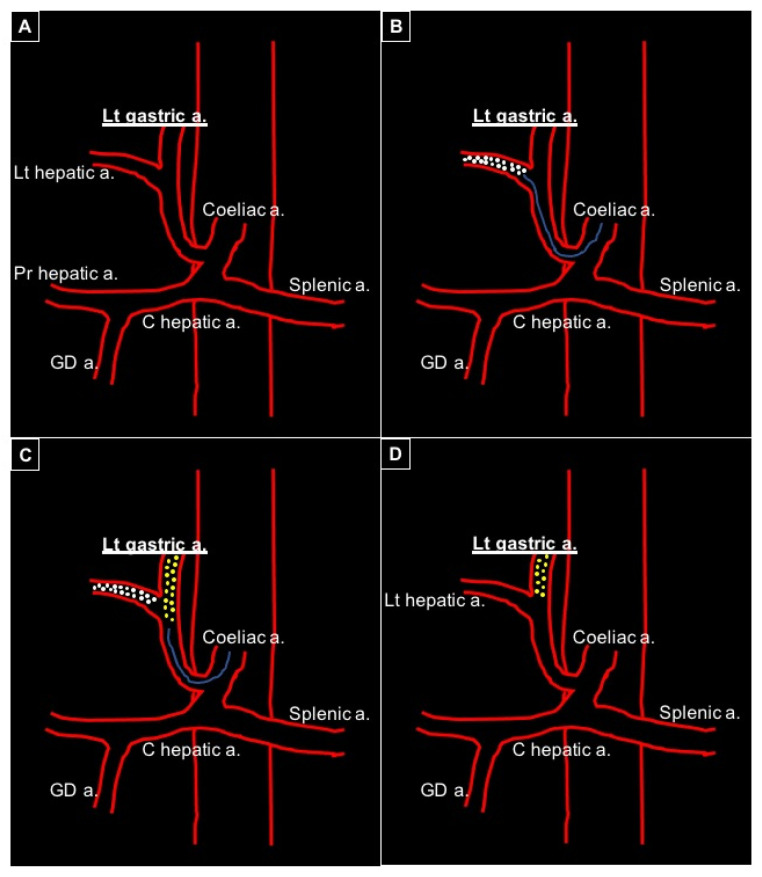
Schematic representation of flow diversion from the left hepatic artery to the left gastric artery in a case of gastrohepatic trunk anatomical variation, with the embolization target being the left gastric artery. (**A**) Gastrohepatic trunk variation: left hepatic artery originating from left gastric artery. (**B**) A microcatheter is positioned via the gastrohepatic trunk into the left hepatic artery and temporary embolization is performed by injection of Embocept-S^®^ microparticles. (**C**) The microcatheter is retrieved proximally, before the bifurcation, and definitive embolization of the left gastric artery is obtained with microparticles. (**D**) Patency of the left hepatic artery is restored, with Embocept-S^®^ microparticles being reabsorbed in 35 min, while the target (left gastric artery) is definitely embolized.

All interventions were conducted via a 5 French (Fr) peripheral access (right femoral or left radial); with a 4 Fr or 5 Fr diagnostic catheter, with different tips (Cobra 1, Sheperd, Simmons 1, Headhunter, Multipurpose) according to the arterial anatomy, the coeliac trunk was selected. A digital subtracted arteriography (DSA) was acquired using a power injector (flow: 4–5 mL/s; volume 15–16 mL; pressure 900 psi) in order to depict the coeliac and mesenteric districts anatomy and planify the embolization procedure (Figure 1A). The main arterial trunk was then selectively engaged with a microcatheter (1.8–2.4 Fr) and additional distal DSA acquisitions were performed to depict the arterial network of the target. In all patients included in this study, the target could not be selected and so non-target adjacent vessels, not sparable from the embolization, were identified. Therefore, to protect the healthy tissues, non-target vessels were selected with the microcatheter and Embocept-S^®^ microparticles were injected up to stop-flow for 5 consecutive heart beats, in order to obtain a temporary flow diversion and exclude the non-target area to the following embolization (Figure 1B).

Once the temporary embolization of the non-target district was obtained, the microcatheter was proximally retrieved and a DSA was acquired to assess the arterial flow diversion to the target district in order to complete the embolization of the target (Figure 1C).

A final DSA confirmed proper embolization of the district (Figure 1D), with the DSMs reabsorbed in 35 min (100% reabsorption, 90 min).

In case of femoral arterial access, a mechanical closure device was positioned to seal the arterial access, and the patient lay supine for 24 h, according to the local protocol [7]; in case of radial access, a bracelet cuffed with air (17–20 mL) was positioned and the patient lay supine for 2 h.

All procedures were performed in an inpatient regimen and under local anesthesia with mild sedoanalgesia. Wide-spectrum antibiotic prohylaxis was performed with 2 g of Cefazolin endovenous.

**Figure 2:**
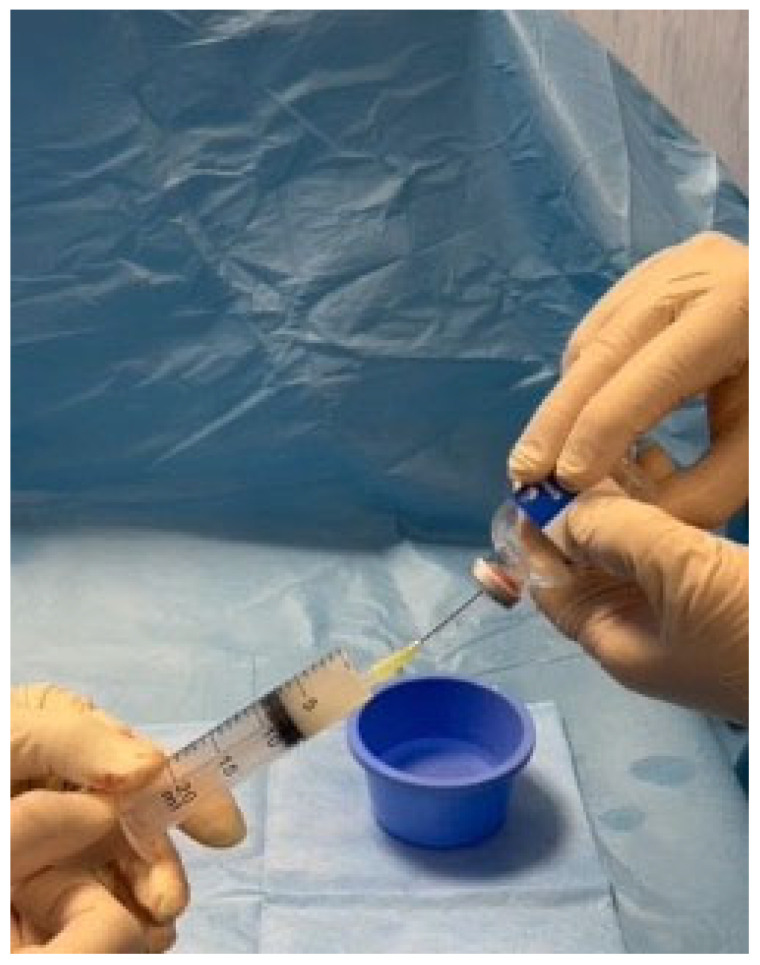
Embocept-S^®^ particles were prepared by aspiring the full content of the Embocept-S^®^ microparticles vial (7.5 mL) in a 20 mL syringe via a 19-gauge needle. Then, the particles were diluted with 12.5 mL of iodinated contrast agent and injected with a small 2.5–3 mL syringe via a microcatheter.

DSMs were prepared as follows: the full content of the Embocept-S^®^ vial (7.5 mL) was aspired in a 20 mL syringe via a 19-gauge needle (Figure 2) and diluted with 12.5 mL iodinated contrast agent in order to obtain radiopacity of the particles; the particles were then aspired in a 2.5 mL or 3 mL syringe to be injected via the microcatheter into the non-target vessel.

### 2.2. Statistical Analysis

All data were collected and classified in an Excel v.16.43 environment (Microsoft Corporation^®^, Redmond, WA, USA).

Descriptive data analyses were performed in an Excel environment; quantitative parameters were expressed with mean values and the range was reported.

## 3. Results

A total of 16 patients were included in this analysis, with a mean age of 68 years (range: 42–81), including 14 males and 2 females.

### 3.1. Procedures and Embolics

**Figure 3:**
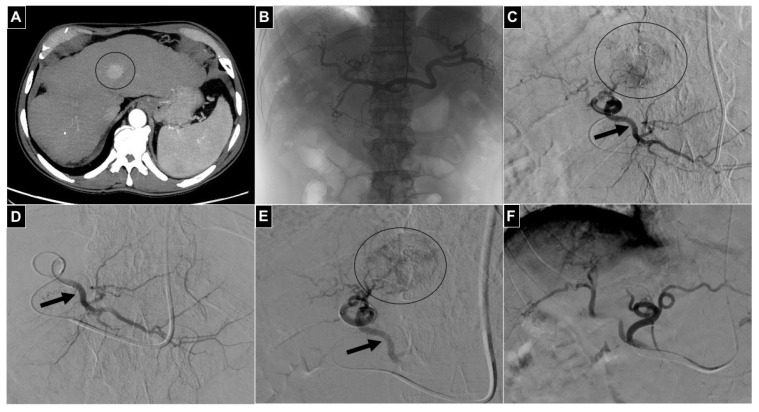
A 61-year-old male with multifocal HCC; treatment with DSM-TACE was selected. (**A**) A 3 cm nodule in segment IVa (black circle) is evident in the CT scan in the arterial phase. (**B**) Selective coeliac trunk arteriography is acquired via a 5 Fr diagnostic catheter positioned via radial access, and normal anatomy is appreciable. (**C**) Left hepatic artery is selected with a 2.4 Fr microcatheter and DSA is acquired; the HCC nodule (black circle) is refurnished by small branches not suitable for microcatheter selection. (**D**) Flow diversion by temporary embolization of segmental branches II-III is performed by advancing the microcatheter tip (black arrow). (**E**) After Embocept-S^®^ particle injection, stop flow into the segmental branches II-III is obtained (black arrow) and arterial flow is solely directed to the HCC nodule (black circle); the microcatheter tip is retrieved proximally and DSM-TACE is performed. (**F**) Final DSA control showing no more nodule opacification in segment IVa.

**Figure 4:**
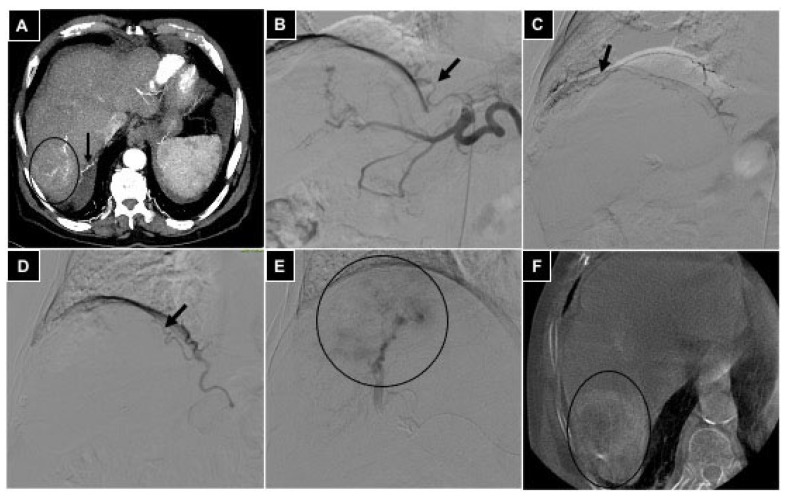
An 81-year-old male with a single 6 cm HCC nodule; treatment with TARE was selected. (**A**) A 6 cm HCC nodule in segment VII (black circle) is evident in the CT scan in the arterial phase; an additional lesion feeding from the right phrenic artery (black arrow) is appreciable. (**B**) During the preliminary work-up procedure, selective coeliac trunk arteriography is acquired via a 5 Fr diagnostic catheter positioned via femoral access, and a hypertrophic right phrenic artery is evident (black arrow). (**C**) A 2.4 Fr microcatheter is advanced into the right phrenic artery and a DSA is acquired, showing nodule refurnishment from small distal branches (black arrow). (**D**) Flow diversion by means of temporary embolization of the right phrenic artery (black arrow) is performed, and stop flow is obtained by Embocept-S^®^ microparticle injection. (**E**) The microcatheter is advanced into the right hepatic artery and a DSA is acquired, showing nodule refurnishment (black circle). (**F**) A ConeBeamCT is acquired before ^99m^Tc MAA injection and target opacification is evident (black circle).

**Figure 5:**
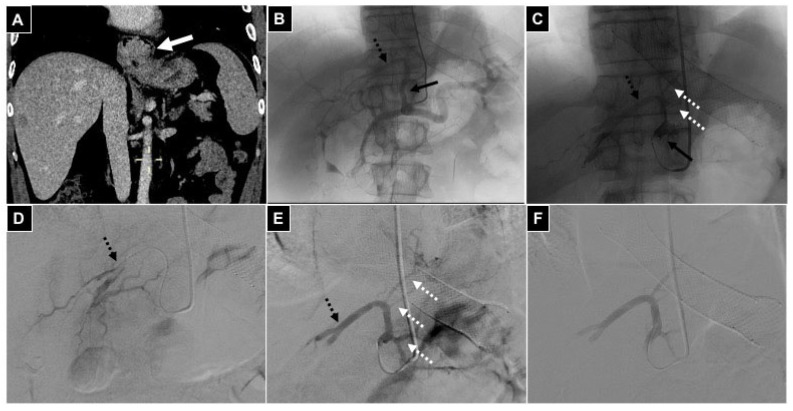
A 42-year-old male with gastric adenocarcinoma involving the cardias region and causing chronic anemia; palliative bland embolization is selected. (**A**) Coronal CT scan in venous phase showing the neoplastic lesion (white arrow), and a liver metastasis is also evident in segment VII. (**B**) Selective coeliac trunk arteriography is acquired via a 5 Fr diagnostic catheter positioned via radial access, and gastrohepatic trunk variation is evident (black arrow), with the left hepatic artery (black dotted arrow) originating from the left gastric artery; a palliative gastrooesophageal metallic stent is appreciable too. (**C**) A 2.4 Fr microcatheter is positioned into the gastrohepatic trunk (black arrow) and DSA is acquired showing small branches refurnishing the tumor (white dotted arrows) not suitable for microcatheterism, and the left hepatic artery (black dotted arrow) is evident. (**D**) The microcatheter is advanced into the left hepatic artery (black dotted arrow). (**E**) After injection of Embocept-S^®^ microparticles, temporary embolization of the left hepatic artery (black dotted arrow) is obtained and arterial flow is diverted to the lesion (white dotted arrows), so embolization with definitive 500–700 μm microparticles is conducted. (**F**) Final DSA control showing no more opacification of left gastric branches and left hepatic artery.

Peripheral embolization procedures were performed in the coeliac visceral district, all in oncologic patients (Table 1), including 4 transarterial radioembolization (TARE) work-ups in patients with hepatocarcinoma (HCC) (Figure 3), 10 TACE procedures in patients with HCC (8) (Figure 4) or cholangiocarcinoma (CCC) (2), and 2 palliative transarterial embolizations (TAE) in patients with gastric cancer (Figure 5).

Among the four patients undergoing TARE work-up, temporary flow diversion was obtained by embolizing the right phrenic artery (two cases) or intrahepatic segmental arteries (two cases) with DSMs, before ^99m^Tc macroaggregated albumin injection. Among the 10 patients treated with TACE, DSM embolization for flow diversion was performed in 8 cases in intrahepatic segmental arteries and in 2 cases in the left gastric artery because the left hepatic originated from it. In the two palliative TAEs for gastric cancer performed to reduce chronic blood loss, the left hepatic artery was temporary embolized with DSM because it originated from the left gastric artery.

In TARE procedures, the target tumor was embolized with ^99m^Tc macroaggregated albumin; in TACE procedures, the target tumor was chemioembolized with conventional TACE, DSM-TACE or DEB-TACE, according to operator preference; and in the palliative TAE, the target tumor was embolized with 500–700 μm microparticles.

### 3.2. Follow-Up

At 30 days, patients were evaluated in an outpatient setting with laboratory data and CT imaging.

Laboratory data were evaluated during the recovery and at 30 days: immediately after the procedure, a slight transient increase (range: 75–133 for GOT; 41–167 for GPT) in transaminasis was observed in 13 patients (81.2%), which actually normalized at 30 days follow-up in all cases; in no cases were these increases five times the preprocedural value; these patients had been treated with TACE, two patients with TARE, and one patient treated with TAE. A slight increase in total bilirubin values was also observed (range: 0.3–0.7) in seven patients (43.7%), immediately after TACE procedures; similarly to transaminasis, these parameters normalized at 30 days follow-up.

The white cell count increased in 14 patients (87.5%), ranging from 10,587 to 16,122/mL; these represented all patients treated with TACE and TAE and two patients treated with TARE, and these values normalized at follow-up

No specific treatment was required for patients with laboratory data, except for hydration.

Regarding CT follow-up, no procedural-related complications were detected; in all cases, the healthy anatomical districts embolized with DSMs were regularly perfused without signs of ischemia.

### 3.3. Procedural Outcomes

Technical success was obtained in 100% of the cases, while clinical success was reached in 87.5%: in two TACE procedures, despite technical success, the procedural clinical benefits were partial, with an incomplete target lesion response at 30 days follow-up CT.

Complications occurred in five patients (31.2%), all of which were minor (grade Ia) and related to post-embolization syndrome (abdominal pain and nausea); these occurred in four patients after TACE and in one patient after gastric cancer embolization.

## 4. Discussion

In this study on 16 patients, temporary flow diversion with Embocept-S^®^ was safe and effective during visceral embolization interventions involving hepatic, gastric and phrenic arteries; in all cases, the target lesion could not be accessed superselectively with the microcatheter and so, to protect healthy surrounding tissues, temporary embolization with DSMs was performed. While no major complications were observed, minor complication in terms of post-embolization occurred in 31.2% of cases; however, it is unclear if they depended on flow diversion or the cancer treatment itself. Furthermore, post-embolization is considered a self-limiting sequela, expected in 30% of the cases of TACE according to literature data [5] as an effect of an efficient liver-directed procedure. Similarly, transient increases in transaminasis, bilirubin, and white cell count were observed in the majority of the cases, but these values normalized at 30 days follow-up without additional therapies. However, it is possible that these laboratory findings were related to the embolization of the target itself rather than the flow diversion embolization; indeed, it has clearly been demonstrated that after TACE and TARE procedures, as well as all visceral embolizations, transient effects on liver function and inflammatory reactions, respectively, are common sequelae [8,9,10,11]. Interestingly, no ischemia of the healthy districts treated with DSMs were noted in 30 days follow-up CT, confirming the transient embolic effect of the particles; this result should also be considered in the light of the exclusion criteria (previous surgery in the treated district), because post-surgical variations in the arterial network together with neovascularization processes could occur, and in such cases, embolization effects could not be predictable.

Flow diversion is usually performed via definitive stenting of the target district in cases of aneurysm repair; this approach is largely applied in intracranial aneurysm embolization [1], while in peripheral visceral aneurysm procedures, encouraging data have emerged recently [12,13,14,15]. In some cases, flow diversion has been performed via the embolization of non-target vessels with coils, plugs, or glue, especially during early TARE interventions [16,17,18,19,20], but this approach entails the definitive occlusion of arteries refurnishing non-target healthy tissue. However, when only temporary flow diversion is required, as in the case of oncological embolizations, flow diverter stents are not applicable, and so different approaches are required. Previously published experiences have been reported in the case of TARE procedures to redirect the arterial flow to the target lesion and sparing healthy tissues: Abdelsalam et al. [21] reported temporary flow diversion in an HCC TARE of segment 4 by deploying a microvascular plug without detaching to protect hepatic segmental branches for segments 2 and 3. Other colleagues have described the similar application of a balloon microcatheter in liver TACE and TARE to redirect the arterial flow to the lesion when the target vessel could not be selected with the microcatheter [22,23,24] or to redirect arterial flow to a single feeder [25]. However, with both plugs and balloons, a second microcatheter is needed, and this implies upsizing the guiding sheath to accommodate two microcatheters within it or provide access to a second artery; furthermore, these procedures increase costs and are time consuming compared ti the technique proposed in this paper with temporary degradable microparticle injection.

Another embolic agent that could be considered for a similar purpose thanks to its temporal effect is gelfoam; however, multiple concerns regarding its use should be analyzed. For instance, the particle size is not calibrated and this leads to unpredictable occlusion levels, while injection rates with slurry increase risks for reflux to the tumor vasculature [26], which ultimately could result in a compromised tumor treatment. In addition, its resorption time varies from days to weeks [27].

Interestingly, Young et al. [28] distinguish flow diversion from flow redistribution: while the first indicates the blockade of adjacent healthy liver tissues to protect them from non-target deposition during TACE or TARE, flow redistribution means the embolization of competing tumor extrahepatic vascular supply in order to stimulate tumor perfusion from the intrahepatic feeders; in their paper, the authors successfully performed flow diversion with both definitive (100–500 μm microsphere and microcoils) and temporary embolics (gelfoam, temporary balloon occlusion, and a retrieved microvascular plug).

Nowadays, only a small case-series has described the use of DSMs to perform temporary flow diversion: Meyer et al. [29] reported success in five patients receiving DSM temporary embolization of healthy tissues before TARE for HCC. Similarly to our study, these authors performed protective temporary embolization using DSMs of normal liver tissue that could not be excluded from the area treated by TARE through catheter repositioning. No adverse events associated with DSM occurred, neither relevant increases in transaminasis or bilirubin values; the authors therefore concluded that temporary embolization with DSM before radioembolization is feasible and can effectively protect areas of normal liver tissue from irradiation and avoid permanent embolization.

In this series, DSM flow diversion has been effectively performed not only during liver cancer treatment with TACE or TARE but also in case of palliative embolizations of gastric cancer; this demonstrates that the application spectrum of this technique is diverse and multiple technical scenarios, not only oncological procedures but also acute hemorrhages, for example, could benefit from this approach.

Finally, operators must provide careful fluoroscopy visualization of the particles by properly mixing the contrast agent with DSM and keeping the particles suspended; this approach, together with gentle injection, is mandatory to avoid reflux to the target tumor, thus compromising its final embolization.

This study presents some limitations: first of all, the overall number of patients is small and larger studies involving high-volume centers are required to confirm the proposed data [30]; the sample is heterogeneous with different types of tumor and organs involved—however, this could also demonstrate the feasibility of this approach in multiple scenarios, even extrahepatic; and finally, a follow-up to confirm the full patency of the vessels temporarily embolized with DSMs is lacking—however, the starch particles’ degradation in 40 min has been already confirmed [31,32].

## 5. Conclusions

In conclusion, in this study, temporary flow diversion with DSMs during oncological embolization procedures was safe and effective; this approach is suitable for protecting healthy surrounding tissues when the vessels feeding the target cannot be accessed superselectively with the microcatheter.

## Figures and Tables

**Table 1:** Procedural details.

Procedures	Underlying Diseases	Non-Target Vessels Temporary Embolized with Embocept-S^®^	Procedural Target Vessels	Embolic Agents to the Target
4 TARE	HCC	2 Phrenic a. 2 Intrahepatic segmental a.	Intrahepatic a.	^99m^Tc MAA
10 TACE	8 HCC 2 CCC	8 Intrahepatic segmental a. 2 Left gastric a.	Intrahepatic a.	Lipiodol-doxorubicin Preloadable chemiotherapic beads Embocept-S^®^-doxorubicin
2 TAE	Gastric cancer	2 Left hepatic a.	Left gastric a.	Bland microparticles

TARE: transarterial radioembolization; TACE: transarterial chemioembolizations; TAE: transarterial embolization; HCC: hepatocarcinoma; CCC: colangiocarcinoma; a.: artery; Tc: tecnetium; MAA: macroaggregated albumin.

## Data Availability

The data presented in this study are available upon request from the corresponding author (francescogiurazza@hotmail.it). The data are not publicly available for the privacy protection of the patients involved, in accordance with the current law of the country in which the study was conducted.
